# Turn down - turn up: a simple and low-cost protocol for preparing platelet-rich plasma

**DOI:** 10.6061/clinics/2019/e1132

**Published:** 2019-08-13

**Authors:** Edilson Silva Machado, Renata Leite, Cintia Cichowski dos Santos, Georgia Lazzari Artuso, Fernando Gluszczak, Leonardo Giovani de Jesus, José Manuel Peixoto Caldas, Markus Bredemeier

**Affiliations:** I Servico de Dor e Cuidados Paliativos, Grupo Hospitalar Conceicao, Hospital Nossa Senhora da Conceicao, Porto Alegre, RS, BR; II Centro de Pesquisa e Gerenciamento da Dor, Clinica Univida, Canoas, RS, BR; III Laboratorio de Analises Clinicas, Grupo Hospitalar Conceicao, Hospital Nossa Senhora da Conceicao, Porto Alegre, RS, BR; IV Public Health Institute, Medical School, Universidade do Porto, Porto, Portugal; V Servico de Reumatologia, Grupo Hospitalar Conceicao, Hospital Nossa Senhora da Conceicao, Porto Alegre, RS, BR

**Keywords:** Platelet-Rich Plasma, Regenerative Medicine, Orthopedics

## Abstract

**OBJECTIVE:**

To describe and analyze a new protocol for the extraction of platelet-rich plasma (PRP) for use in clinical practice and compare this technique with methods that have been previously described in the medical literature.

**METHODS:**

We extracted PRP from 20 volunteers using four different protocols (single spin at 1600 ×g, single spin at 600 ×g, double spin at 300 and 700 ×g, and double spin at 600 and 900 ×g). In another group of 12 individuals, we extracted PRP with our new technique (named ‘turn down-turn up') consisting of a double spin (200 ×g and 1600 ×g) closed system using standard laboratory equipment (including an ordinary benchtop centrifuge), where the blood remained in the same tube during all processes, reducing the risk of contamination. Platelet counts adjusted to baseline values were compared using analysis of covariance (ANCOVA).

**RESULTS:**

Using the four previously described protocols (mentioned above), we obtained concentrations of platelets that were 1.15-, 2.07-, 2.18-, and 3.19-fold greater than the baseline concentration, respectively. With the turn down-turn up technique, we obtained a platelet count that was 4.17-fold (95% confidence interval (CI): 3.09 to 5.25) greater than the baseline platelet count (*p*=0.063 compared with the double spin at 600 and 900 ×g method). The total cost of the disposable materials used in the extraction process was less than US$10.00 per individual.

**CONCLUSION:**

In the present study, we described a simple and safe method for obtaining PRP using low-cost devices.

## INTRODUCTION

The incidence of articular degenerative diseases is increasing 1, resulting in a substantial economic and social burden 2. Even with advancements in understanding the biological mechanisms of joint diseases, a therapy that controls or reverses the degenerative process is not yet available in clinical practice. Surgical procedures (arthroplasties and arthrodesis) that are associated with high costs and a high rate of complications remain the definitive options for these types of lesions 3,4. The development of low-cost and minimally invasive therapies for orthopedics has led to the use of biological therapies, especially platelet-rich plasma (PRP), to optimize clinical outcomes and improve tissue recovery 5. Studies on the therapeutic applications of PRP have been conducted in several fields, such as maxillofacial surgery 6, cardiovascular surgery 7, and plastic surgery 8. In orthopedics, the clinical indications include hip and knee osteoarthritis, rotator cuff pathologies 8, epicondylitis and tendinitis 9, fracture healing 10, and back pain 11. A recent meta-analysis pooled the results of clinical trials of PRP injection in patients with knee osteoarthritis and demonstrated that PRP is superior to intra-articular injections of corticosteroids and hyaluronic acid 12.

PRP is defined as an autologous preparation obtained from the centrifuged peripheral blood of patients with a concentration that is 3-5 times greater than the basal concentration 13. Through the separation of blood components (plasma, platelets, leukocytes and red blood cells) by centrifugation, platelets are located in the lower portion of the plasma layer next to the hematic region. There has been increasing interest in the therapeutic effects of PRP upon the demonstration of its potential tissue repair ability. Growth factors present in platelets are responsible for a regenerative stimulus. Vascular endothelial growth factor (VEGF), platelet-derived growth factor AB (PDGF-AB), and transforming growth factor beta 1 (TGF-b1) are several growth factors released by platelets that are responsible for tissue repair 14. Cell proliferation, angiogenesis, and cell migration are stimulated by these growth factors, resulting in tissue repair and regeneration 15,16. The number of platelets per volume of blood varies between individuals, and the expression of growth factors is also not uniform 17. There is still no absolute definition of the number of platelets that is required for a regenerative stimulus. Ideally, the platelet concentration should be close to 1,000,000 platelets/mL, and studies showing the best results in bone healing reported a platelet concentration that is 3 to 8 times greater than the basal concentration 18.

The first step to carry out a clinical study on PRP is the standardization of the process for collecting and preparing the concentrate. Several previous studies have reported different methods of obtaining PRP using different centrifugation speeds and numbers of spins 11. Appropriate care during collection and manipulation and control of temperature are also important to ensure that the early activation of platelets does not occur 19. Some investigators have proposed standard protocols for evaluating the quality of PRP, mainly for reproducibility purposes and for the inclusion of studies in systematic reviews and meta-analyses 20. In general, PRP preparation in clinical studies involves complex and expensive protocols, and the majority of studies do not provide sufficient information to allow for adequate reproducibility. A detailed, precise, and stepwise description of the PRP preparation protocol is required to allow comparisons between studies.

In the present study, we aimed to describe and evaluate a simple, inexpensive, and reproducible protocol for obtaining PRP for use in clinical practice.

## MATERIALS AND METHODS

The first part of the study involved the reproduction of four different protocols based on a literature review. Healthy blood donors were selected from a hospital blood bank. After explaining the research objectives to patients and obtaining signed informed consent forms from the patients, 42.5 ml of peripheral venous blood was obtained from each patient. For coagulation analysis, blood was collected using blood collection tubes (BD Vacutainer ACD Solution A Ref. 364606) containing 3.2% sodium citrate at a volumetric ratio of 1.5 ml of anticoagulant to 8.5 ml of blood. A Sorologic Kasvi centrifuge (model K14-0815A) with a fixed angle rotor was used.

The blood samples were analyzed on a Sysmex XE5000 hematology counter prior to the centrifugation procedures to establish the baseline values of platelets, red blood cells, and leukocytes in each patient. After obtaining the concentrate, a new count was obtained to enable the evaluation of the platelet concentration factor (the ratio between platelet counts in the concentrate and the original blood sample). The extraction of PRP was performed following four different protocols. Protocol 1 was used to obtain samples based on commercial kits mentioned in previous studies 21,22, and the samples were centrifuged at 1600 ×g for 3 minutes, while samples obtained by protocol 2 23 were centrifuged at 600 ×g for 5 minutes. Both protocols involved single spin methods. Protocols 3 and 4, which were double spin methods, consisted of centrifugation at 600 ×g 24,25 and 300 ×g 16,26,27 for 5 minutes. After careful collection of 2 ml of the lower portion of the plasma while avoiding aspirating the buffy coat, the material was again centrifuged at 900 ×g for 15 minutes (protocol 3) or at 700 ×g for 17 minutes (protocol 4).

In the second part of the study, we investigated our protocol (‘turn down-turn up') for obtaining PRP. The procedures are described in Box 1. The estimated time for preparation of PRP using this protocol was approximately 35 minutes.

**Turn Down-Turn Up PRP Protocol - Double Spin - Closed System:**

**Figure 1:**
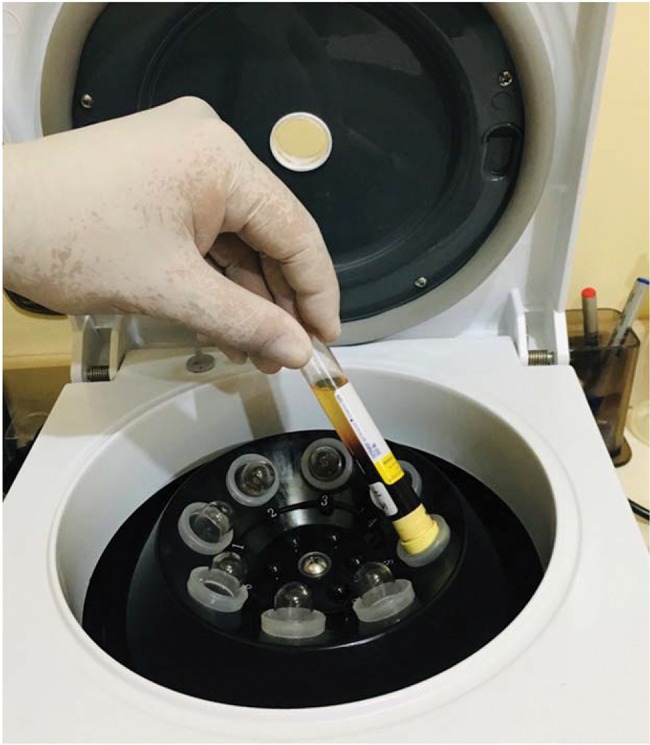
The tube must be inserted with the cap facing down.

**Figure 2:**
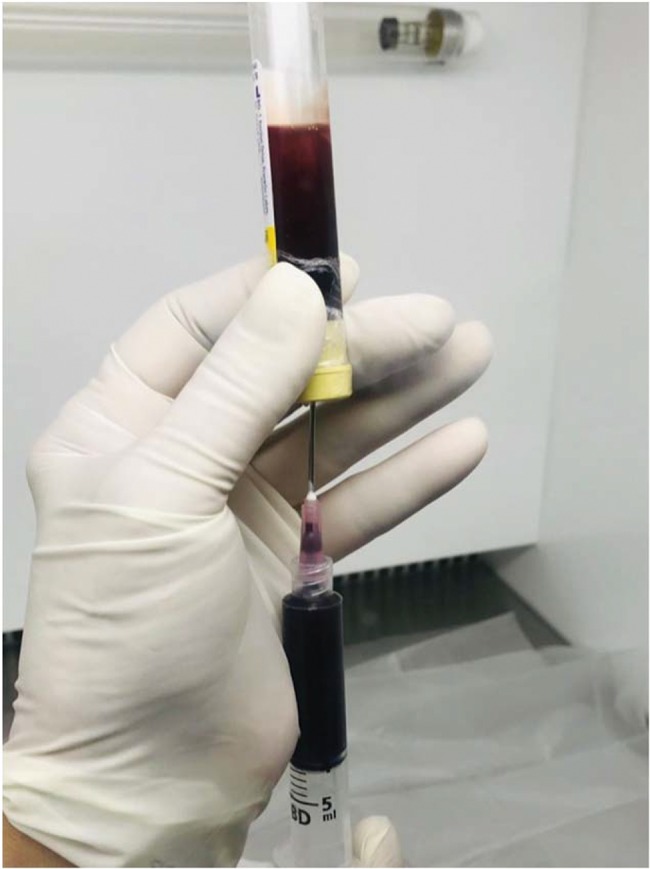
Aspiration of the hematic layer with the cap facing down.

**Figure 3:**
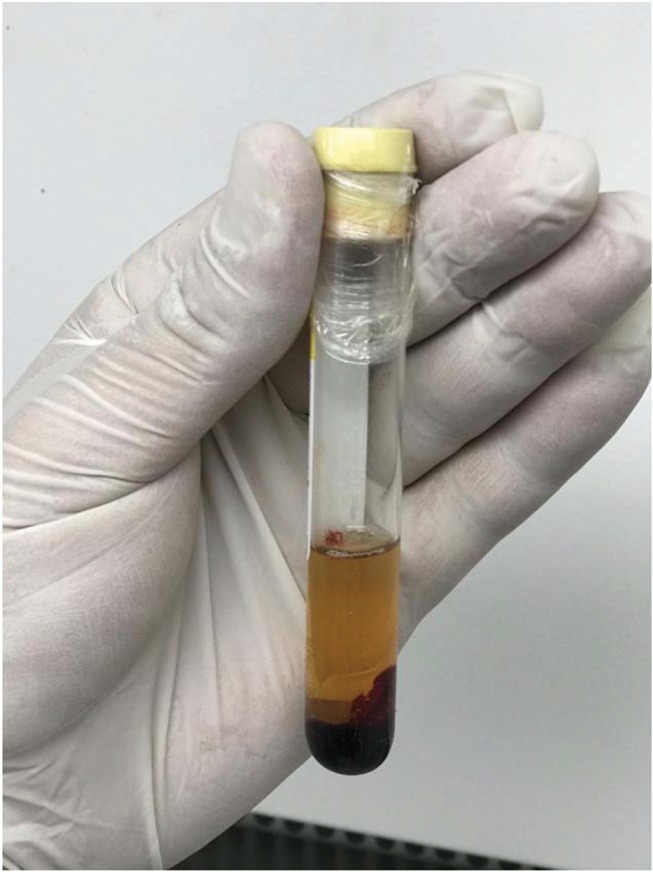
The final appearance of the tube after the second spin.

1. Collect the desired volume (8.5 ml) of blood through peripheral venous access directly into a vacuum tube with acid citrate dextrose (ACD) (1.5 ml).

2. Equalize the remaining vacuum in the tube.

3. Centrifuge the tube at 200 ×g for 15 minutes with the tube cap facing down (Figure 1).

4. Carefully remove the tube from the centrifuge and maintain the tube in the downward position without turning the tube.

5. Under aseptic conditions, aspirate 3.5 ml of the hematic layer through the rubber cap (Figure 2).

6. Turn the tube to an upright position (cap facing up).

7. Centrifuge the tube at 1600 ×g for 10 minutes with the lid facing up.

8. Under aseptic conditions, aspirate 3.5 ml of the superior portion of the material (platelet-poor plasma, PPP).

9. Aspirate the desired amount of PRP (1-2 ml) from the lower portion of the tube (Figure 3).

### Statistical analysis

The platelet counts were compared with Student's *t*-test and analysis of covariance (adjusted for baseline platelet counts) using IBM SPSS Statistics for Windows, Version 20.0 (IBM Corporation, Armonk, NY, USA). The 95% confidence intervals (CIs) were estimated for the ratio between the PRP and baseline concentrations.

### Ethics

The present study was approved by the Institutional Review Board of our institution (Hospital Nossa Senhora da Conceição, Grupo Hospitalar Conceição) and registered at Plataforma Brasil (the Brazilian government's registry of scientific studies) under CAAE (‘Certificado de Apresentação de Apreciação Ética', Certificate of Presentation of Ethic Appreciation) number 53166416.1.0000.5530.

## RESULTS

For the first part of the study (the investigation of previously described protocols), 20 male donors aged 26 to 54 years (mean age: 39.9 years, standard deviation (SD): 9.9 years) were selected. The baseline counts of the blood components were within the normal range of values (Table 1).

The PRP extraction results are described in Table 1. For the single spin protocols, the mean final concentration of platelets was 1.17 times greater than the basal concentration with the 1600 ×g protocol and 2.15 times greater than the basal concentration with the 600 ×g protocol. The 300 ×g+700 ×g protocol yielded a platelet concentration that was 2.21 times greater than the basal concentration. A higher concentration was obtained with the 600 ×g+900 ×g protocol that was 3.09 times greater than the basal concentration (Table 1).

In the second part of the study (the investigation of the turn down-turn up PRP protocol developed by our team), another group of donors (10 males and 2 females) was selected (mean age: 38.7 years, SD: 9.9 years, range: 28-54 years). Our protocol achieved a mean platelet concentration that was 4.07 times greater than the basal concentration with a mean platelet count of 749x10^3^ platelets/μL (Table 1). This platelet concentration tended to be greater than that obtained with the 600 ×g+900 ×g protocol (Student's t-test, *p*=0.059; ANCOVA adjusted for the baseline platelet count, *p*=0.063). Analysis of the PPP revealed negligible values (values ranging from 1 to 16 x 10^3^ cells/uL) of red and white blood cells. The number of platelets and erythrocytes lost at the end of the first centrifugation was also evaluated, and the median count was 48.75 x 10^3^ platelets/μL (26.4% of the baseline platelet count), which varied from 29 to 66 x 10^3^ platelets/μL. Therefore, an average of 73.6% of the total number of platelets was recovered by our PRP preparation protocol.

## DISCUSSION

The use of PRP in various medical fields has resulted in the exponential growth of the number of studies using PRP therapy. In orthopedics, PRP serves as an intermediate between conservative and surgical treatments 28. The diversity of protocols causes difficulties in comparing and/or reproducing results 13. Chahla et al. conducted a systematic review of studies investigating the use of PRP for the treatment of orthopedic pathologies and indicated that only 10% of these studies provided comprehensive reporting that included a clear description of the preparation protocol. They concluded that a detailed, precise, and stepwise description of the PRP preparation protocol is required to allow comparisons between studies and enable reproducibility 11. The need for consensus on the minimum reporting requirements for studies evaluating biologic treatments has been suggested by the American Academy of Orthopedic Surgeons. After a think tank symposium, a consensus of experts was published listing the minimum data that should be reported in studies to favor standardization of the analyses 29. The minimum information for clinical studies evaluating biologics in orthopedics (MIBO statement) is available at the following web address: www.mibo-statement.org. Some authors proposed methods of PRP classification. A more comprehensive and comparative investigative strategy has been proposed by Lana et al. 30: the MARSPILL classification describes the method (custom-made or commercial kit), activation, presence of red and white blood cells, number of spins, platelet concentration and number, light activation and image guidance of the application. This new classification focuses on mononuclear cells, which are as important as the platelet content due to their action on neovasculogenesis and cellular proliferation.

In addition to the lack of standardization of the methods to obtain PRP, the frequency and number of PRP injections remain controversial. In previous studies, the protocols for injections are highly variable, with up to 6 weekly applications in the lumbar muscles for the treatment of back pain 11. Furthermore, there is evidence that a single injection in an arthritic knee could be enough 31. In Brazil, the Federal Medical Council currently recognizes PRP as a promising therapy, but its use is officially permitted only in clinical protocols that are duly evaluated and approved by the CEP/CONEP system. The main argument is the lack of standardization of PRP preparation and lack of information regarding the optimal dose and frequency of applications 32.

In the present study, we performed PRP extraction using four methods adapted from previously reported protocols. We obtained a low volume of platelet concentrate using single spin protocols in accordance with previous reports. Cavallo et al. 31 compared different formulations of PRP extraction and found a significant difference between the single and double spin protocols The protocols using double spins were also investigated in this study but yielded heterogeneous results. Centrifugation at 300 ×g followed by 700 ×g achieved an intermediate platelet concentration (2.18 times greater than the baseline concentration). However, in the original study conducted by Amable et al. 16, who obtained platelets at concentrations up to 7 times greater than the baseline concentration, all steps were performed in a refrigerated centrifuge with temperatures ranging from 8 to 19°C. The use of a refrigerated centrifuge is justified because the plasma density and viscosity differ at lower and higher temperatures 16. Since we aimed to identify a reproducible protocol for easy clinical use, the experiments that we did were conducted at room temperature (22°C), and the centrifuges were not refrigerated.

Among the previously established protocols investigated in this study, the highest concentrations of platelets were obtained using the method reported by Jo et al. 24. In their original study, they achieved 633 x 10^3^ platelets/uL, which was 4.3 times greater than the basal concentration of platelets, with a nonrefrigerated centrifuge. Using this method, we obtained a platelet concentration that was 3.09 times greater than the basal concentration. This difference may be explained by several factors, which we briefly discuss here. The results may have been influenced by the design of the centrifuge. Rotors with a fixed/nonfixed angle and the distance to the center of the rotor may result in different forces, leading to different concentration values, and the size of the blood tube may also influence the results 25.

There is still no consensus or scientific basis for the concentration of platelets that is necessary to induce tissue repair. However, there is evidence that even relatively low platelet concentrations may be effective. Sanchez et al. found that, compared with hyaluronic acid injection in the knee, using the single spin technique, which produced a concentration of platelets that was two-fold greater than the baseline concentration, showed a superior effect 33. Furthermore, most investigators suggest concentrations that are 4-5 times greater than the baseline concentration. A study conducted by Sugaya et al. 34 comparing PRP and bone marrow aspirate concentrate (BMAC) showed a wide range of basal platelet and growth factor concentrations between research volunteers.

After reviewing the current literature and investigating four different techniques of PRP extraction, we developed a reproducible and low-cost closed protocol, which we named the turn down-turn up technique. This two-spin method follows the principles of low-speed centrifugation to preserve the maximum volume of platelets and growth factors 34,35 and uses discontinuous centrifugation to modulate and control platelet recovery. According to Perez et al., the centrifugal acceleration, time, size of the rotor, volume of processed blood, and prevention of platelet aggregation are the most relevant factors that must be controlled in the centrifugation step for the preparation of PRP 27. Their maximum platelet recovery was observed after centrifugation at 100 ×g for 10 minutes in 3.5 ml tubes. Therefore, we adapted the first spin at 200 ×g for 15 minutes considering that the size of the tube used by us was 10 mL. We analyzed the volume of platelets lost in the hematic portion, and the values were within an acceptable range according to previous studies. The very low number of platelets and white blood cells found in the PPP after the second centrifugation confirmed the high degree of platelet recovery of our technique. We estimated that our cost of PRP preparation in a hospital facility is less than US$10.00 without considering the expenses of equipment and laboratory personnel. Various other centrifuge-based closed systems, such as PRP kits, are available commercially with a price ranging between US$300.00 to $1,500.00 20. Our technique has the advantages of a closed system but with less cost and a low risk of contamination.

## CONCLUSIONS

In the present study, we described a simple and safe method of obtaining PRP using low-cost devices. Our results were consistent with current standards of quality for PRP preparation for clinical trials suggested by the American Academy of Orthopedic Surgeons working group, who presented the MIBO statement 3. Compared with other currently employed techniques, our method may have significant advantages. The proposed technique does not demand expensive laboratory kits or a refrigerated centrifuge and has a low risk of contamination considering the use of a closed system.

## AUTHOR CONTRIBUTIONS

Machado ES provided substantial contribution to the concept and design of the study, data acquisition, analysis and interpretation, manuscript drafting, critical revision for important intellectual content and final approval. Leite R, Santos CC, Artuso GL provided substantial contribution to the concept and design of the study, data acquisition, manuscript critical revision for important intellectual content and final approval. Caldas JM provided substantial contribution to the concept and design of the study, manuscript critical revision for important intellectual content and final approval. Gluszczak F provided substantial contribution to data acquisition, analysis and interpretation, manuscript critical revision for important intellectual content and final approval. Jesus LG provided substantial contribution to the data analysis and interpretation, manuscript drafting and final approval. Bredemeier M provided substantial contribution to the data analysis and interpretation, manuscript drafting, critical revision for important intellectual content and final approval. All of the authors take public responsibility for appropriate portions of the content. 

## Figures and Tables

**Table 1:** Results of the platelet counts using the different PRP extraction techniques that were investigated in this study.

First part of the study (the investigation of previously described protocols; N=20).					
	Baseline	1600 ×g (Protocol 1)	600 ×g (Protocol 2)	300+700 ×g (Protocol 3)	600+900 ×g (Protocol 4)
Mean platelet count±SD (range of values)*	188.8±28.9 (152 to 248)	225.4±162.8 (17 to 524)	394.0±87.8 (202 to 524)	412.5±83.0 (249 to 594)	599.9±41.5 (355 to 1081)
Fold change relative to the baseline value±SD (95% CI)	------------	1.15±0.77 (0.813 to 1.49)	2.07±0.30 (1.94 to 2.2)	2.18±0.29 (2.05 to 2.31)	3.19±0.91 (2.79 to 3.59)
**Second part of the study (the investigation of the turn down-turn up technique); N=12.**					
	**Baseline**	**Turn down-turn up**			
Mean Platelet count±SD (range of values)*	185.4±27.9 (152 to 225)	749.0±307.0 (426 to 1395)			
Fold change relative to the baseline value±SD (95% CI)	----------	4.17±1.9 (3.09 to 5.25)			

* values represent thousands of platelets per μL. CI: confidence interval.
